# Ligand Effects on Intramolecular Configuration, Intermolecular Packing, and Optical Properties of Metal Nanoclusters

**DOI:** 10.3390/nano11102655

**Published:** 2021-10-09

**Authors:** Sainan Wu, Xiao Wei, Hao Li, Honglei Shen, Jiaojiao Han, Xi Kang, Manzhou Zhu

**Affiliations:** Department of Chemistry and Centre for Atomic Engineering of Advanced Materials, Key Laboratory of Structure and Functional Regulation of Hybrid Materials of Ministry of Education, Institutes of Physical Science and Information Technology and Anhui Province Key Laboratory of Chemistry for Inorganic/Organic Hybrid Functionalized Materials, Anhui University, Hefei 230601, China; WSN_chem@163.com (S.W.); weixiao_chem@163.com (X.W.); speechless95@outlook.com (H.L.); shenhonglei_chem@163.com (H.S.); hjj_chem@163.com (J.H.)

**Keywords:** atomically precise nanoclusters, surface modification, intramolecular configuration, intermolecular packing, optical properties

## Abstract

Surface modification has served as an efficient approach to dictate nanocluster structures and properties. In this work, based on an Ag_22_ nanocluster template, the effects of surface modification on intracluster constructions and intercluster packing modes, as well as the properties of nanoclusters or cluster-based crystallographic assemblies have been investigated. On the molecular level, the Ag_22_ nanocluster with larger surface steric hindrance was inclined to absorb more small-steric chlorine but less bulky thiol ligands on its surface. On the supramolecular level, the regulation of intramolecular and intermolecular interactions in nanocluster crystallographic assemblies rendered them CIEE (crystallization-induced emission enhancement)-active or -inactive nanomaterials. This study has some innovation in the molecular and intramolecular tailoring of metal nanoclusters, which is significant for the preparation of new cluster-based nanomaterials with customized structures and enhanced performances.

## 1. Introduction

Metal nanoclusters, bridging between small-sized molecular complexes and large-sized plasmonic nanoparticles, have attracted considerable attention owing to their atomically precise structures and excellent electrical, optical, and catalytical properties directed by the discrete electronic energy level as well as the structure-dependent quantum confinement effect [1,2,3,4,5,6,7,8,9,10,11,12,13,14,15,16]. The monodispersed sizes, precise compositions, and accurate configurations of metal nanoclusters make it possible to investigate the relationship between their structures and properties. In addition, the attainable structure–property correlations further enable the rational construction of new nanoclusters with customized performances [17,18,19,20,21,22]. In this context, the regulatable intramolecular structures and intermolecular packing modes render metal nanoclusters or cluster-based nanocomposites prominent nanomaterials for atomic engineering and further practical applications [23,24,25,26,27,28,29].

The past few decades have witnessed great research efforts of the control over intracluster structures/compositions and intercluster aggregates [17,18,19,20,23,24,25,26,27]. Specifically, the intramolecular control of nanoclusters touches upon the manipulation of their metal-ligand compositions and bonding environment at the single molecular level, while the intermolecular control of nanoclusters refers to the manipulation over their aggregating patterns among several cluster molecules in amorphous or crystallographic forms [30]. Several control methods, including (i) intracluster approaches (e.g., ligand exchange [31,32,33,34], heteroatom alloying [35,36,37,38,39], and molecular charge regulation [40,41,42]) and (ii) intercluster approaches (e.g., cluster-based metal-organic framework [43,44,45,46], aggregation-induced emission [47,48,49], and intercluster metallophilic reaction [50,51]), have been exploited to control clusters or their assemblies and to dictate their properties. Of note, the intracluster and intercluster controls are not a binary separation, but an interrelated and inseparable whole to regulate the nanocluster system simultaneously. In this context, the intracluster regulation of nanoclusters may alter their aggregating patterns at the supramolecular level, and vice versa [52]. The profound cognition of the correlation between molecular and supramolecular chemistry of nanoclusters offers great opportunities for the fabrication of novel nanoclusters or cluster-based hybrids with customized properties.

Herein, a new Ag_22_ nanocluster, formulated as Ag_22_(S-Adm)_10_(DPPM)_4_Cl_6_ (abbreviated as **Ag_22_-L1**, where S-Adm = 1-adamantanethiol and DPPM = bis(diphenylphosphino)methane), was synthesized and structure-determined by X-ray single-crystal diffraction. The combination of this Ag_22_ nanocluster and a previously reported Ag_22_(SPhMe_2_)_12_(DPPE)_4_Cl_4_ (abbreviated as **Ag_22_-L2**, where SPhMe_2_ = 2,5-dimethyl thiophenol and DPPE = 1,2-bis(diphenylphosphino)ethane) constructed a platform to investigate the effects of surface modification on intramolecular constructions and intermolecular packing modes, as well as the properties of nanoclusters or cluster-based crystallographic assemblies. On the molecular level, because of the larger surface steric hindrance of **Ag_22_-L1** relative to **Ag_22_-L2**, the **Ag_22_-L1** surface contained more small-steric chlorine but fewer bulky thiol ligands. On the supramolecular level, **Ag_22_-L2** displayed intramolecular and intermolecular interactions in its crystallographic assembly, while these interactions were absent in the **Ag_22_-L1** crystal. **Ag_22_-L2** was CIEE (crystallization-induced emission enhancement) active while **Ag_22_-L1** was CIEE inactive. The optical absorptions and emissions of these two Ag_22_ nanoclusters were also compared.

## 2. Materials and Methods

**Chemicals.** All reagents were purchased from Adamas Reagent (Shanghai, China) and used without further purification: silver nitrate (AgNO_3_, 99%, metal basis), 1-adamantanethiol (HS-Adm, 97%), 2,5-dimethyl thiophenol (HS-PhMe_2_, 97%), bis(diphenylphosphino)methane (Ph_2_P-CH_2_-PPh_2_, DPPM, 98%), 1,2-bis(diphenylphosphino)ethane (Ph_2_P-C_2_H_5_-PPh_2_, DPPE, 98%), sodium cyanoborohydride (NaBCNH_3_, 99.9%), methylene chloride (CH_2_Cl_2_, HPLC grade), methanol (CH_3_OH, HPLC grade), ethyl ether ((C_2_H_5_)_2_O, HPLC grade), and *n*-hexane (Hex, HPLC grade).

**Synthesis of Ag_22_(S-Adm)_10_(DPPM)_4_Cl_6_ (Ag_22_-L1).** Specifically, 60 mg of AgNO_3_ (0.36 mmol) and 40 μL of H_2_PtCl_6_ (0.2 g/mL; 0.015 mmol) were dissolved in 20 mL of CH_3_OH and 1 mL of CH_3_CN. Then, 40 mg of DPPM (0.1 mmol) and 30 mg of HS-Adm (0.18 mmol) were added. After stirring for 30 min, 100 mg of NaBCNH_3_ (1.59 mmol; dissolved in 2 mL of MeOH) was added. The reaction was allowed to proceed for 5 h. After that, the mixture in the organic phase was rotavaporated under vacuum and washed several times by MeOH and Hex. Then, 10 mL of CH_2_Cl_2_ was used to extract the obtained **Ag_22_-L1** nanocluster. The yield is 30% based on the Ag element (calculated from AgNO_3_). Of note, although Pt did not exist in the final **Ag_22_-L1**, the absence of Pt sources resulted in the failure of the nanocluster synthesis (Figure S1). Such a phenomenon has also been observed in previous works [53]. 

**Synthesis of Ag_22_(S-PhMe_2_)_12_(DPPE)_4_Cl_4_ (Ag_22_-L2).** The preparation of Ag_22_(S-PhMe_2_)_12_(DPPE)_4_Cl_4_ was based on the reported method of the Pradeep group [54].

**Crystallization of Ag_22_-L1.** In order to accelerate the crystallization process and improve the crystal quality, the counterions (i.e., Cl^−^) in the **Ag_22_-L1** nanocluster were replaced by SbF_6_^−^ [55]. The reaction equation was [Ag_22_(S-PhMe_2_)_12_(DPPE)_4_Cl_4_]Cl_2_ + 2 SbF_6_^−^ → [Ag_22_(S-PhMe_2_)_12_(DPPE)_4_Cl_4_](SbF_6_)_2_ + 2Cl^−^. Nanoclusters were crystallized in a CH_2_Cl_2_/ether system with a vapor diffusion method (Table S1).

## 3. Results

The **Ag_22_-L1** nanocluster was synthesized by directly reducing the Ag-SR-DPPM complexes by NaBCNH_3_ (Scheme S1; see more details in *Materials and Methods*). The electrospray ionization mass spectrometry (ESI-MS) measurement was performed to verify the molecular composition and to determine the valence state of the **Ag_22_-L1** nanocluster. As shown in Figure S2, the mass result of the nanocluster exhibited an intense peak at 2897.54 Da. The excellent match of the experimental and simulated isotope patterns illustrated that the measured formula was [Ag_22_(S-Adm)_10_(DPPM)_4_Cl_6_]^2+^. The “+2” valence state of the nanocluster matched well with the existence of (SbF_6_)^−^ counterions in the crystal lattice, i.e., the molar ratio between the cluster and the counterion was 1:2, as depicted in Figure S3. According to the valence states of **Ag_22_-L1**, its nominal electron counts was determined as 4e [56], i.e., 22(Ag) − 10(SR) − 6(Cl) − 2(charge) = 4e, the same as that of **Ag_2_-L2** [54]. Moreover, the chlorine ligands in **Ag_22_-L1** were proposed to originate from the H_2_PtCl_6_ or from the CH_2_Cl_2_ solvent, which has also been discovered in previously determined nanoclusters [57,58,59,60].

**Figure 1:**
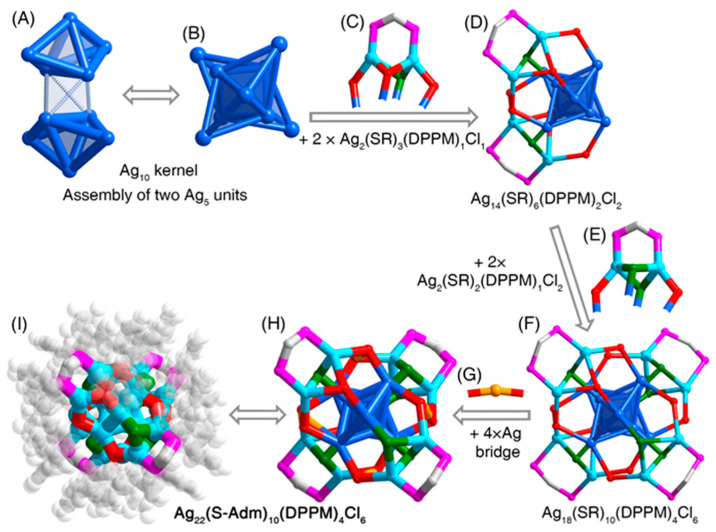
**Structural anatomy of the Ag_22_-L1 nanocluster.** (**A**,**B**) The Ag_10_ kernel, constituted by assembling two Ag_5_ units. (**C**) Two Ag_2_(S-Adm)_3_(DPPM)_1_Cl_1_ surface units. (**D**) The Ag_14_(S-Adm)_6_(DPPM)_2_Cl_2_ structure. (**E**) Two Ag_2_(S-Adm)_2_(DPPM)_1_Cl_2_ surface units. (**F**) The Ag_18_(S-Adm)_10_(DPPM)_4_Cl_6_ structure. (**G**) Four Ag bridges. (**H**,**I**) Overall structure of the Ag_22_(S-Adm)_10_(DPPM)_4_Cl_6_ nanocluster. Color codes: blue/light blue/orange sphere, Ag; red sphere, S; magenta sphere, P; green sphere, Cl; grey sphere, C; white sphere, H.

Structurally, the **Ag_22_-L1** nanocluster contained an Ag_10_ kernel which comprised two distorted trigonal bipyramidal Ag_5_ units via an edge–edge vertical assembling mode (Figure 1A,B). Then, two Ag_2_(S-Adm)_3_(DPPM)_1_Cl_1_ surface units capped the Ag_10_ kernel from the same side via Ag-S or Ag-Cl interactions, giving rise to an Ag_14_(S-Adm)_6_(DPPM)_2_Cl_2_ structure (Figure 1C,D). The other unprotected side of the Ag_10_ kernel was further stabilized by two Ag_2_(S-Adm)_2_(DPPM)_1_Cl_2_ surface units, making up a Ag_18_(S-Adm)_10_(DPPM)_4_Cl_6_ structure (Figure 1E,F). Finally, four Ag atoms acting as bridges linked these surface units via S-Ag-S interactions, yielding the final Ag_22_(S-Adm)_10_(DPPM)_4_Cl_6_ framework (Figure 1G,H). Because of the asymmetry of surface units in **Ag_22_-L1**, especially the asymmetrical arrangement of peripheral thiol and chlorine ligands, no symmetrical element was observed in the **Ag_22_-L1** nanocluster framework (Figure 1I and Figure S4).

**Figure 2:**
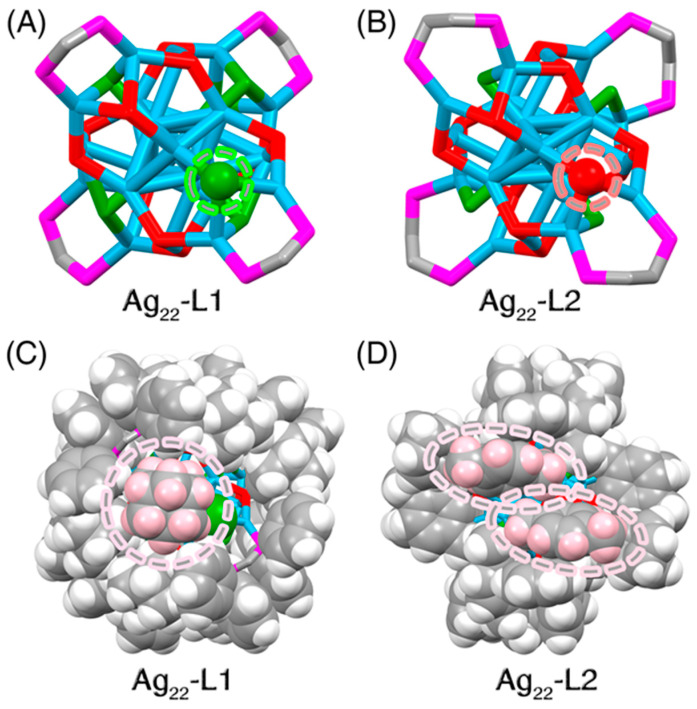
**Structure comparison between Ag_22_-L1 and Ag_22_-L2 nanoclusters.** (**A**) Cluster framework of the **Ag_22_-L1** nanocluster with Cl ligands at specified locations. (**B**) Cluster framework of the **Ag_22_-L2** nanocluster with SR ligands at specified locations. (**C**) Spacefill packing of the **Ag_22_-L1** nanocluster with a S-Adm ligand at the specified surface vacancy. (**D**) Spacefill packing of the **Ag_22_-L2** nanocluster with two S-PhMe_2_ ligands at the specified surface vacancy. Color codes: light blue sphere, Ag; red sphere, S; magenta sphere, P; green sphere, Cl; grey sphere, C; pink/white sphere, H.

The overall constructions of **Ag_22_-L1** and **Ag_22_-L2** nanoclusters were almost the same. However, because of the different steric hindrances of ligands in these two nanoclusters (i.e., S-Adm and DPPM in **Ag_22_-L1**; S-PhMe_2_ and DPPE in **Ag_22_-L2**), these two nanoclusters displayed some structural differences:

(i) For the kernel structure: the average Ag-Ag bond length in bipyramidal Ag_5_ of **Ag_22_-L1** was 2.824 Å, much shorter than that in **Ag_22_-L2** (i.e., 2.933 Å). In addition, the average Ag-Ag bond lengths between these two Ag_5_ bipyramids were 2.870 and 2.937 Å in **Ag_22_-L1** and **Ag_22_-L2**, respectively. In this context, due to the larger surface steric hindrance of **Ag_22_-L1** relative to **Ag_22_-L2**, the Ag_10_ kernel of the former nanocluster was compressed.

(ii) For the surface environment: the biggest structural difference between the two Ag_22_ nanoclusters lay in their surface ligand environments in terms of the proportion of the chlorine in peripheral ligands. Specifically, the **Ag_22_-L1** nanocluster contained 10 thiol and 6 chlorine ligands, while **Ag_22_-L2** included 12 thiol and 4 chlorine ligands (Figure 2). As shown in Figure 2A,B, a thiol ligand at the specific location on the **Ag_22_-L2** surface was substituted by a chlorine ligand in **Ag_22_-L1**. Another thiol ligand at the symmetrical position was also replaced by chlorine. Such a substitution from bulky thiol to small-steric chlorine was reasonable by considering that the more compact surface environment on **Ag_22_-L1**, resulting from the bulkier DPPM and S-Adm ligands relative to DPPE and S-PhMe_2_, was unable to host as many bulky thiol ligands as **Ag_22_-L2** (Figure 2C,D). Moreover, several intramolecular noncovalent C-H···π and π···π interactions were observed in the **Ag_22_-L2** structure, which was advantageous to the compact packing of its surface ligands [54]. By comparison, none of such noncovalent interactions was observed in **Ag_22_-L1**, which might be another reason that more small-steric chlorine but fewer bulky thiol ligands were arranged on the **Ag_22_-L1** nanocluster surface.

**Figure 3:**
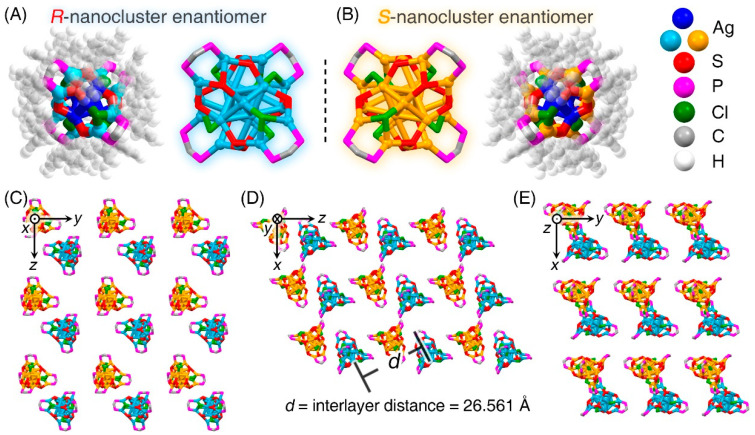
**Crystalline packing of the Ag_22_-L1 nanocluster molecules.** (**A**) Structure of the *R*-nanocluster enantiomer. (**B**) Structure of the *S*-nanocluster enantiomer. (**C**–**E**) Packing of the **Ag_22_-L1** molecules in the crystal lattice: view from the *x* axis (**C**), *y* axis (**D**), and *z* axis (**E**). The inter-layer distance along with the *z* axis is 25.561 Å. Color codes: blue/light blue sphere, Ag in *R*-nanocluster enantiomer; blue/orange sphere, Ag in *S*-nanocluster enantiomer; red sphere, S; magenta sphere, P; green sphere, Cl; grey sphere, C; white sphere, H.

The **Ag_22_-L1** cluster entities were crystallized in a triclinic crystal system with a *P*-1 space group, whereas the **Ag_22_-L2** cluster entities were crystallized in a tetragonal crystal system with an *I*4_1_/*a* space group. Both nanoclusters followed a lamellar eutectic packing pattern between *R*-nanocluster and *S*-nanocluster enantiomers in the crystal lattice; however, due to their distinct crystal systems, the interlayer distances were different: 26.561 Å of **Ag_22_-L1**, and 28.957 Å of **Ag_22_-L2** (Figure 3 and Figure S5). Of note, there are equal *R*-nanocluster and *S*-nanocluster enantiomers in the crystal lattice, and the crystalline material of the nanocluster was racemic. Furthermore, owing to the existence of several benzene-rings in the **Ag_22_-L2** nanoclusters, strong intracluster and intercluster interactions occurred, including C-H···π interaction and π-π stacking [54]. In vivid contrast, these interactions were absent within the **Ag_22_-L1** nanocluster or among **Ag_22_-L1** cluster entities (Figure S6).

**Figure 4:**
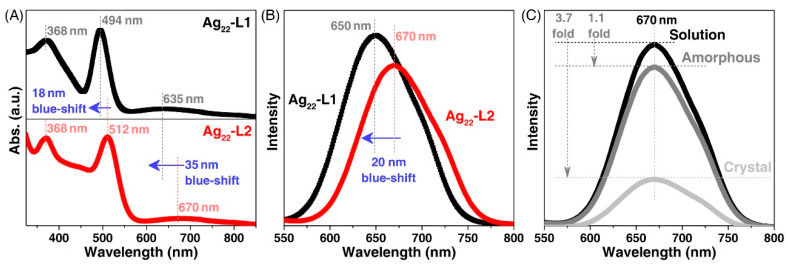
**Comparison of optical properties between two Ag_22_ nanoclusters.** (**A**) Comparison of optical absorptions between **Ag_22_-L1** (black line) and **Ag_22_-L2** (red line). (**B**) Comparison of emissions between **Ag_22_-L1** (black line) and **Ag_22_-L2** (red line). (**C**) Emission spectra of **Ag_22_-L1** in the solution (black line), amorphous (red line), and crystalline (blue line) states.

The **Ag_22_-L1** nanocluster (dissolved in CH_2_Cl_2_) exhibited three intense absorptions centered at 368, 494, and 635 nm (Figure 4A). By comparison, the UV-vis spectrum of **Ag_22_-L2** displayed several peaks at 445, 512, and 670 nm (Figure 4A). The blue shifts in the optical absorptions of **Ag_22_-L1** relative to **Ag_22_-L2** resulted from the different electronic structures of the two Ag_22_ nanoclusters. The CH_2_Cl_2_ solution of **Ag_22_-L1** emitted at 650 nm, while the emission of **Ag_22_-L2** was located around 670 nm (Figure 4B). The 20 nm blue-shift and 1.2-fold enhancement of the emission of **Ag_22_-L1** relative to that of **Ag_22_-L2** resulted from their different electronic structures. Indeed, these two nanoclusters displayed different optical absorptions, demonstrating their distinguishable electronic excitations and HOMO-LUMO energy gaps (HOMO: the highest occupied molecular orbital; LUMO: the lowest unoccupied molecular orbital). In addition, the different electronic excitations endowed these two nanoclusters with distinct emissions.

The **Ag_22_-L2** nanocluster was CIEE active owing to the presence of extensive intramolecular and intermolecular interactions in its crystal lattice [54]. In this context, the emission intensity of **Ag_22_-L2** in the crystalline state was remarkably higher than that of the nanocluster in the solution or the amorphous state. By comparison, the **Ag_22_-L1** was CIEE inactive since no significant enhancement in emission intensity was observed (Figure 4C). Actually, the **Ag_22_-L1** in the amorphous or crystalline state was almost non-emissive. Such a striking contrast was reasonable considering that the intramolecular and intermolecular interactions were absent in the crystal lattice of **Ag_22_-L1**, as mentioned above. The investigation of the Ag_22_ nanocluster system promoted the understanding of the crystalline packing mode and the CIEE of cluster-based nanomaterials.

## 4. Conclusions

In summary, a new Ag_22_ nanocluster, formulated as Ag_22_(S-Adm)_10_(DPPM)_4_Cl_6_, has been synthesized and structurally determined, which constituted an Ag_22_ cluster system together with the previously reported Ag_22_(S-PhMe_2_)_12_(DPPE)_4_Cl_4_. Based on this Ag_22_ cluster system, the effects of surface modification on intracluster constructions and intercluster packing modes, as well as the properties of nanoclusters or cluster-based crystallographic assemblies were investigated. The Ag_22_ nanocluster with larger surface steric hindrance was inclined to load more small-steric chlorine but fewer bulky thiol ligands on its surface. Moreover, the Ag_22_ nanocluster, which embodied several intramolecular and intermolecular interactions in cluster crystallographic assemblies, was CIEE active; by comparison, the Ag_22_ nanocluster without such interactions was CIEE inactive. This work provides new insight into the surface modification of metal nanoclusters and its effects on intramolecular configuration, intermolecular packing, and optical properties.

## Data Availability

The X-ray crystallographic coordinates for structures reported in this work have been deposited at the Cambridge Crystallographic Data Center (CCDC), under deposition numbers CCDC-2106804. These data can be obtained free of charge from the Cambridge Crystallographic Data Centre via www.ccdc.cam.ac.uk/data_request/cif, which has been mentioned in the article.

## Supplementary Materials

The following are available online at www.mdpi.com/article/10.3390/nano11102655/s1, Scheme S1. Synthetic procedure of the nanocluster; Figure S1. Comparison of optical absorptions of the nanocluster synthesis; Figure S2: ESI-MS result of the [Ag_22_(SPhMe_2_)_12_(DPPE)_4_Cl_4_]^2+^ nanocluster; Figure S3. Crystalline unit cell of the [Ag_22_(SPhMe_2_)_12_(DPPE)_4_Cl_4_](SbF_6_)_2_ nanocluster; Figure S4. Overall structure of the [Ag_22_(SPhMe_2_)_12_(DPPE)_4_Cl_4_](SbF_6_)_2_; Figure S5. Crystal unit of **Ag_22_-L2**; Figure S6. Two adjacent Ag_22_(SPhMe_2_)_12_(DPPE)_4_Cl_4_ nanocluster molecules in the crystal lattice; Table S1. Crystal data and structure refinement for the [Ag_22_(SPhMe_2_)_12_(DPPE)_4_Cl_4_](SbF_6_)_2_ nanocluster.
