# Supplementary material for: Ligand Effects on Intramolecular Configuration, Intermolecular Packing, and Optical Properties of Metal Nanoclusters
Source: Nanomaterials (Basel). 2021 Oct 9;11(10):2655. doi: 10.3390/nano11102655 (PMC8537406; doi:10.3390/nano11102655)
Supplement: Supplementary file 1 [file nanomaterials-11-02655-s001.zip › nanomaterials-1408107-supplementary.pdf]

nanomaterials

Supplementary Materials

Ligand Effects on Intramolecular Configuration, Intermolecular Packing, and Optical Properties of Metal Nanoclusters

Sainan Wu, Xiao Wei, Hao Li, Honglei Shen, Jiaojiao Han, Xi Kang * and Manzhou Zhu *

Department of Chemistry and Centre for Atomic Engineering of Advanced Materials, Key Laboratory of Structure and Functional Regulation of Hybrid Materials of Ministry of Education, Institutes of Physical Science and Information Technology and Anhui Province Key Laboratory of Chemistry for Inorganic/Organic Hybrid Functionalized Materials, Anhui University, Hefei 230601, China; WSN_chem@163.com (S.W.); weixiao_chem@163.com (X.W.); speechless95@outlook.com (H.L.); shenhonglei_chem@163.com (H.S.); hjj_chem@163.com (J.H.)

* Correspondence: kangxi_chem@ahu.edu.cn (X.K.); zmz@ahu.edu.cn (M.Z.)

Characterizations. All UV-vis absorption spectra of nanoclusters dissolved in CH_2Cl_2 were recorded using an Agilent 8453 diode array spectrometer.

Photoluminescence (PL) spectra were measured on a FL-4500 spectrofluorometer with the same optical density of 0.1. The nanocluster amorphous samples towards the PL measurement were prepared by directly drying the CH_2Cl_2 solution of the nanocluster. Of note, the densities of nanocluster amorphous and crystal samples were almost the same. Besides, the PL measurements of nanocluster amorphous or crystal samples were measured by the assistant of cover and slide glasses.

Electrospray ionization mass spectrometry (ESI-MS) measurements were performed by Waters XEVO G2-XS QTof mass spectrometer. The sample was directly infused into the chamber at 5 μL/min. For preparing the ESI samples, nanoclusters were dissolved in CH_2Cl_2 (1 mg/mL) and diluted (*v*/*v* = 1:2) by CH_3OH.

Single-crystal analysis. The data collection for single-crystal X-ray diffraction (SC-XRD) of the Ag_{22}(S-Adm)$_{10}$DPPM$_4$Cl$_6$ nanocluster crystal sample was carried out on Stoe Stadivari diffractometer under nitrogen flow, using graphite-monochromatized Cu Kα radiation (λ = 1.54186 Å). Data reductions and absorption corrections were performed using the SAINT and SADABS programs, respectively. The structure was solved by direct methods and refined with full-matrix least squares on F^2 using the SHELXTL software package. All non-hydrogen atoms were refined anisotropically, and all the hydrogen atoms were set in geometrically calculated positions and refined isotropically using a riding model. All crystal structures were treated with PLATON SQUEEZE, and the diffuse electron densities from these residual solvent molecules were removed. The CCDC number of the Ag_{22}(S-Adm)$_{10}$(DPPM)$_4$Cl$_6$ nanocluster is 2106804.

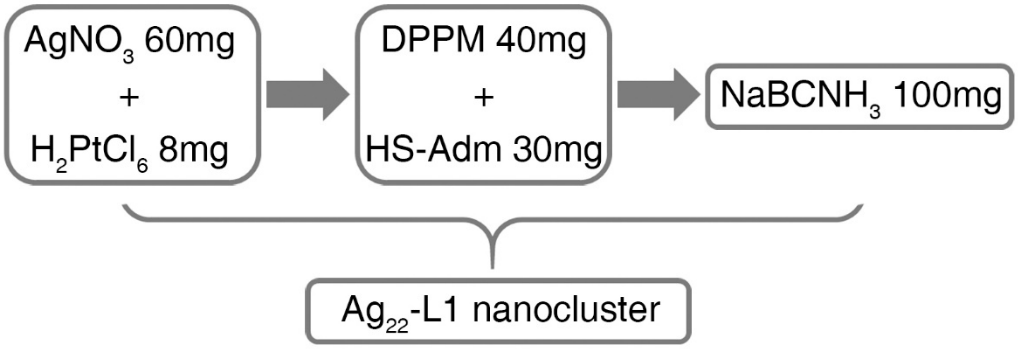

Scheme 1. Synthetic procedure of the nanocluster.

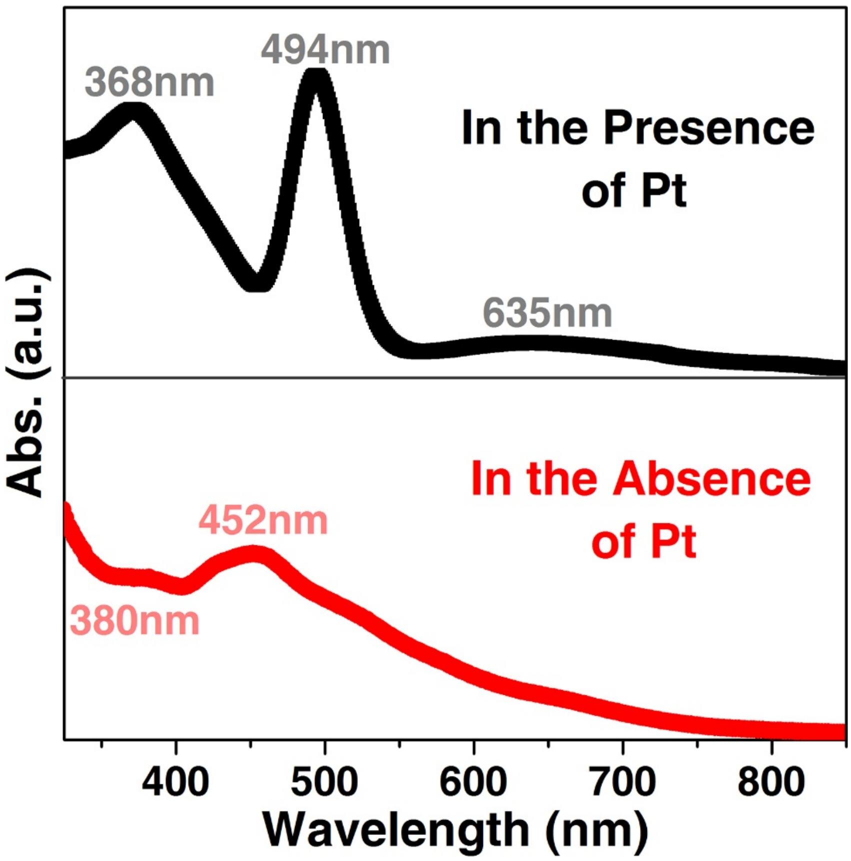

Figure S1. Comparison of optical absorptions of the nanocluster synthesis in the presence of Pt sources (black line) or in the absence of Pt sources (red line). The optical absorption of the product in the absence of Pt sources was totally different from that in the presence of Pt, demonstrating that the absence of Pt sources resulted in the failure of the Ag_{22}-L1 nanocluster synthesis.

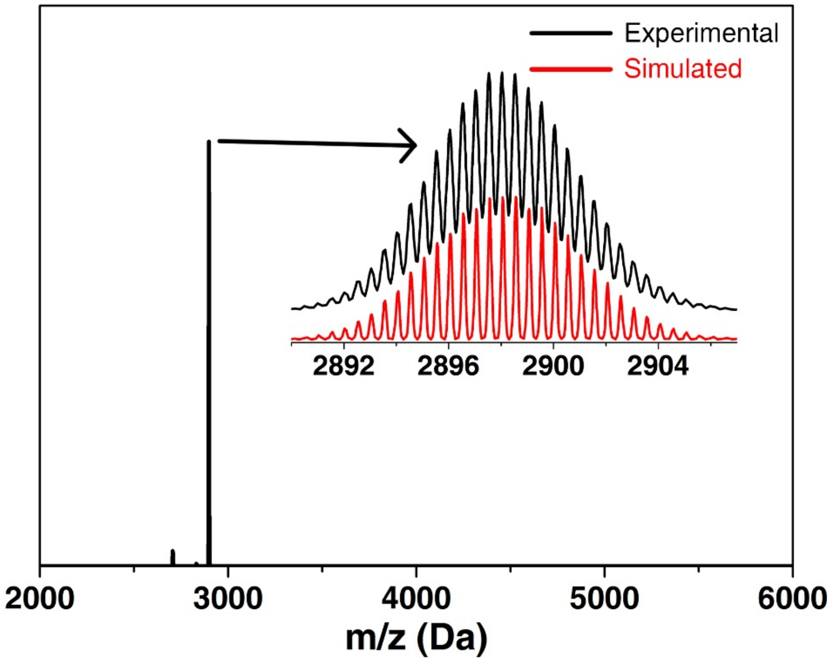

Figure S2. ESI-MS result of the $[Ag_{22}(SPhMe_2)_{12}(DPPE)_4Cl_4]^{2+}$ nanocluster. Insets: the experiment (black line) and the simulated (red line) isotopic distributions.

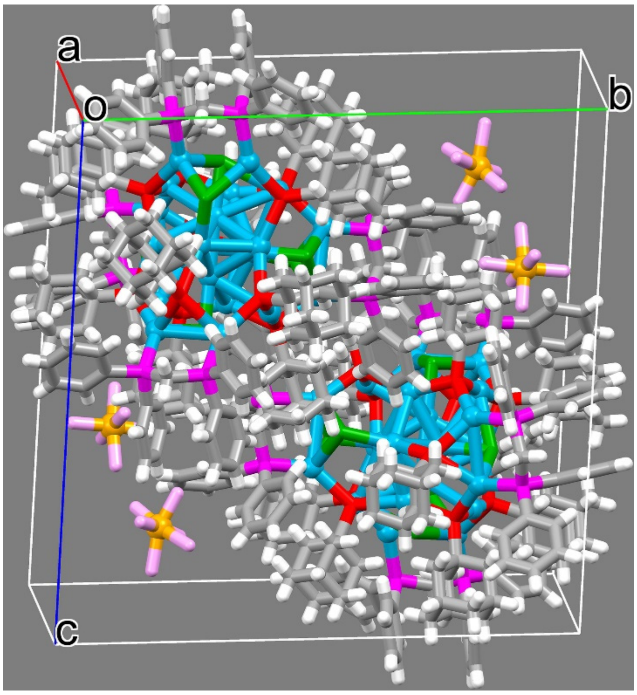

Figure S3. Crystalline unit cell of the $[Ag_{22}(SPhMe_2)_{12}(DPPE)_4Cl_4](SbF_6)_2$ nanocluster. Each nanocluster unit contains two Ag_{22} clusters and four SbF_6 counterions. In this context, the mole ratio between the nanocluster and the counterion is 1:2, demonstrating the "+2" valence state of the nanocluster.

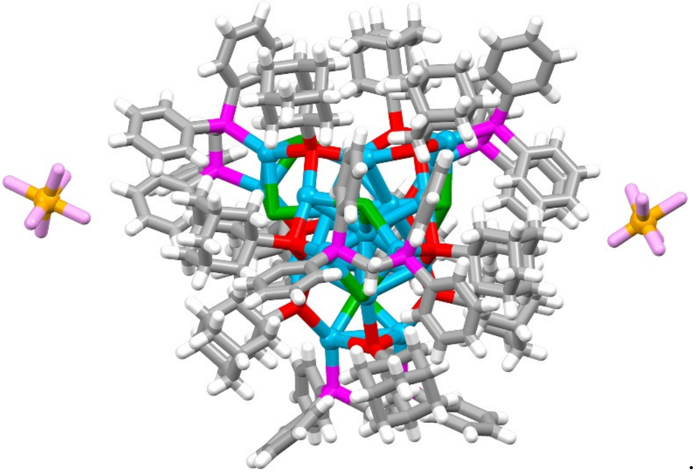

Figure S4. Overall structure of the $[Ag_{22}(SPhMe_2)_{12}(DPPE)_4Cl_4](SbF_6)_2$. No symmetrical element was observed in the nanocluster framework.

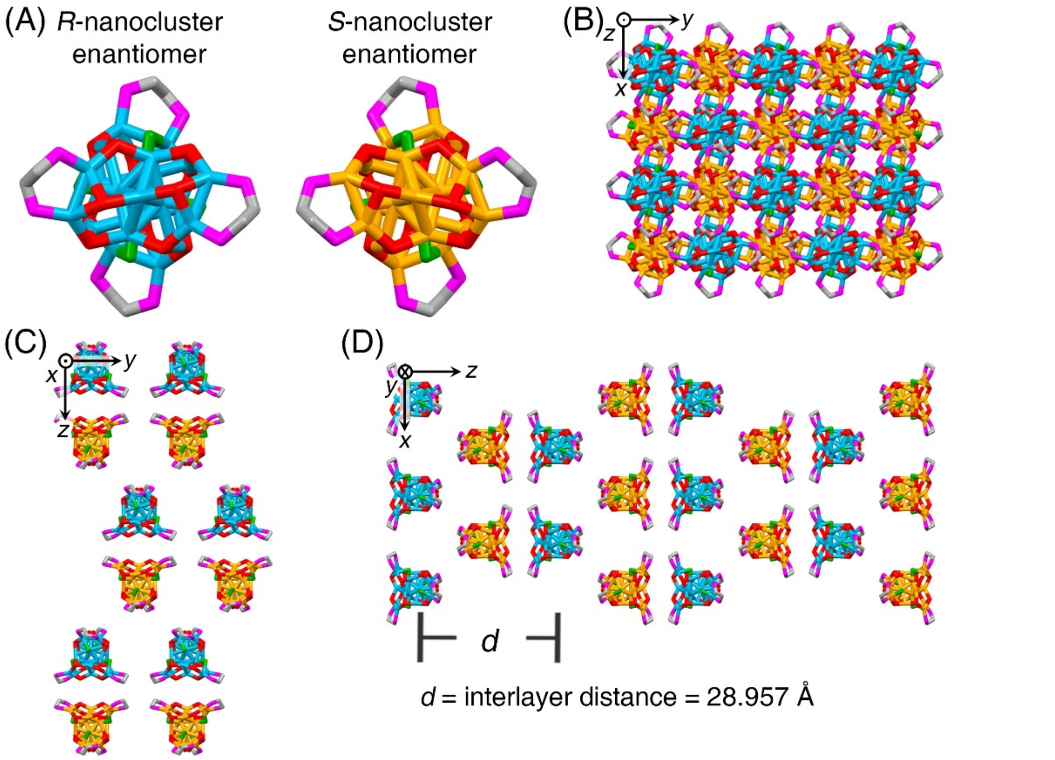

Figure S5. Crystal unit of **Ag$_{22}$-L2**. (**A**) Structures of the *R*-nanocluster enantiomer and the *S*-nanocluster enantiomer of **Ag$_{22}$-L2**. (**B**–**D**) Packing of the **Ag$_{22}$-L2** molecules in the crystal lattice: view from the *z* axis (**B**), *x* axis (**C**), and *y* axis (**D**). The inter-layer distance along with the *z* axis is 28.957 Å.

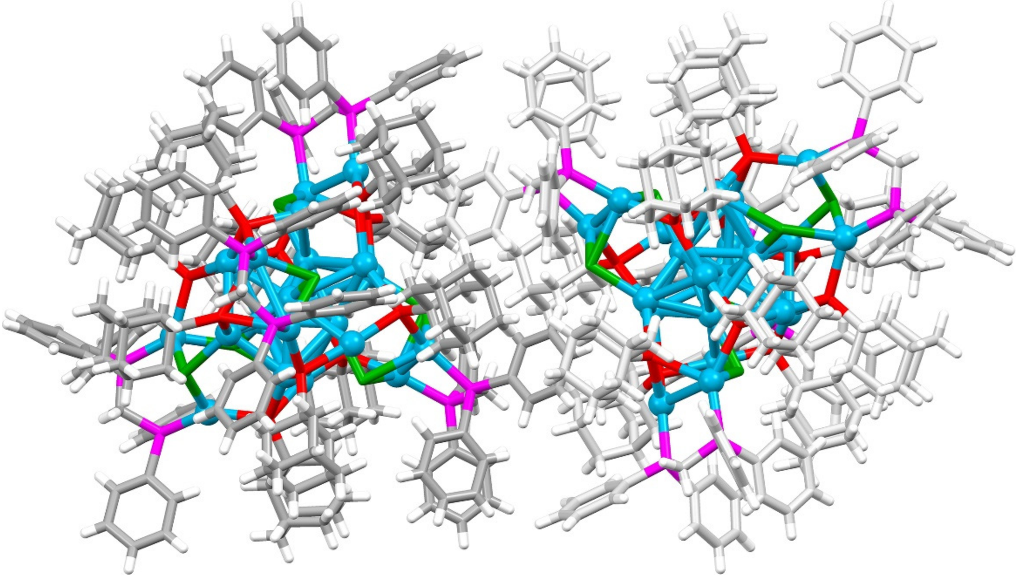

Figure S6. Two adjacent $Ag_{22}(SPhMe_2)_{12}(DPPE)_4Cl_4$ nanocluster molecules in the crystal lattice. No intracluster and intercluster interaction (such as C–H···π interaction and π-π stacking) occurs between these two cluster molecules.

Table S1. Crystal data and structure refinement for the $[Ag_{22}(SPhMe_2)_{12}(DPPE)_4Cl_4](SbF_6)_2$ nanocluster. CCDC number is 2106804.

**Crystal system**	**triclinic**
Space group	*P*-1
a/Å	20.0411(3)
b/Å	24.2609(3)
c/Å	26.5610(4)
α/°	89.2050(10)
β/°	71.6490(10)
γ/°	82.8780(10)
Volume/Å^3	12158.7(3)
Z	2
ϱcalcg/cm^3	1.712
μ/mm^{-1}	17.892
F(000)	6128
Radiation	CuKα (λ = 1.54186)
Index ranges	-23 ≤ h ≤ 9, -27 ≤ k ≤ 27, -30 ≤ l ≤ 26
Final R indexes [I>=2σ (I)]	R1 = 0.0644, wR2 = 0.1770
Final R indexes [all data]	R1 = 0.0861, wR2 = 0.1899
